# Changes in metabolic energy measures for daily living activities and exercise in men and women following arduous activity in Antarctica

**DOI:** 10.1371/journal.pone.0335735

**Published:** 2025-10-31

**Authors:** John Hattersley, C. Doug Thake, Chris Imray, Adrian J. Wilson

**Affiliations:** 1 Human Metabolic Research Unit, University Hospitals Coventry and Warwickshire NHS Trust, Coventry, United Kingdom; 2 School of Engineering, University of Warwick, Coventry, United Kingdom; 3 Centre for Physical Activity, Sport and Exercise Sciences, Coventry University, Coventry, United Kingdom; 4 Department of Vascular and Renal Transplant Surgery, University Hospitals Coventry and Warwickshire NHS Trust, Coventry, United Kingdom; 5 Global Polar and Altitude Metabolic Research Registry, Royal Geographical Society, London, United Kingdom; 6 Department of Physics, University of Warwick, Coventry, United Kingdom; University of Missouri, UNITED STATES OF AMERICA

## Abstract

This study reports pre- to post-expedition (pre|post) changes in energy expenditure and substrate utilisation during daily living activities (DLAs including rest, sleep, modest exercise, sedentary work and leisure) and maximum aerobic power ($\dot{\text{V}}\text{O2max}$) for participants in the Inspire-22 expedition (6 men, 3 women) who undertook a 47 day unassisted Antarctic traverse from the Ronne Ice Shelf to the South Pole. DLA measurements were carried out during a 36 hour study in a whole-body calorimeter whilst measurements of maximum oxygen uptake ($\dot{\text{V}}\text{O2max}$), capillary glucose and lactate during incremental stepping exercise to volitional exhaustion were carried out under normobaric normoxic and hypoxic (14% O2) conditions in an environmental chamber. Non-exercise measures were normalised to non-fat tissue weight; exercise measures, including those in the DLAs, to body weight. Statistical analysis used the Aligned Rank Transform (ART) non-parametric ANOVA technique with covariants pre|post expedition, sex and hypoxia|normoxia as appropriate. There were no systematic differences between pre- and post-expedition energy expenditure but significant differences between men and women for the majority of the DLAs (p < 0.05). There was increased carbohydrate utilisation post-expedition for sleep and the two lower intensity exercise levels (p < 0.05) but not during rest or the highest intensity exercise (p > 0.05); a sex-independent subset of 4 showing much higher utilisation. Women had a lower protein utilisation than men during the DLA exercise activities (p < 0.05). $\dot{\text{V}}\text{O2max}$ was lower in hypoxia than normoxia (p < 0.001) and reduced glucose and lactate between pre- and post-expedition measures. There was also a significant interaction between sex and pre|post measurements for $\dot{\text{V}}\text{O2max}$ and lactate suggesting that adaptation to the environment and high workloads was different between men and women; a suggestion supported by the difference in fat-free tissue measured pre- and post-expedition, but neither sex showed major differences between the pre- and post-expedition measurements.

## Introduction

A prolonged energy deficit is commonly seen in participants in extreme sports [1,2], exploratory journeys [3,4] and military training [5]. Modest energy deficit is an accepted part of these activities that is seen to reverse on return to a normal environment for both sexes [6]. This return to pre-expedition values is assumed to indicate no long-term metabolic consequences. Physiological changes to the challenges presented, for example the elevation of haemoglobin concentration to compensate for altitude [7] and the activation of brown fat to support non-shivering thermogenesis [8] form part of adaptation to the environment. Conversely reversal of these changes following the end of an extreme activity form de-adaptation from the environment. The time over which adaptation occurs is change and environment dependant with activation of brown fat occurring over a period of 10 days [8] whilst elevation of haemoglobin concentration occurs over a small number of days [7]. De-adaptation is much less well studied but typically occurs over a couple of weeks [9,10]. Polar journeys are typically much longer than the adaptation period and travel logistics mean that detailed UK based metabolic measurements are typically made more than 4 weeks before the start of, and after the end of the expedition. Therefore, differences between pre- and post-expedition measurements reflect underlying changes due to the expedition. Recent whole-body calorimeter studies on trans-Antarctic polar expedition participants have shown little difference in pre- to post-expedition energy expenditure and substrate utilisation for either men or women with the possible exception of a sex-independent increase in carbohydrate utilisation in a subset of participants when fasted, but not when fed [6]. In all participants the weight loss was modest indicating appropriate and adequate nutrition during the expedition. In this context, some studies on male subjects [11,12] have shown a change in metabolic pathways to prioritise glucose utilisation rather than protein when exposed to moderate altitudes. However, another study showed a reduction in glucose utilization in male subjects, albeit when exposed to a lower altitude [13]. No change was seen in glucose utilization in women exposed to similar altitudes [14].

Previous studies of changes to adverse environments have often been based on single sex measurements, including changes due to Antarctic exploratory journeys [6]. Where mixed sex groups have been studied, results are not always partitioned on sex [15]. In this study we report whole-body calorimetry and exercise measurements on a mixed sex group who undertook a Transantarctic crossing so comparisons between the sexes can be made directly.

Indirect whole-body calorimetry determines the metabolic rate of a subject [16,17] undertaking a variety of activities including rest, sleep, sedentary activities (e.g., reading, writing, watching video material) and modest exercise to a standard protocol [3,4] over extended periods. The components of the protocol can be considered Daily Living Activities (DLAs) which we distinguish from Activities of Daily Living (ADLs), the tasks used by Occupational Health and other professionals to assess independent living capability. The typical day during the expedition for those undertaking polar exploratory journeys will probably include much higher levels of physical activity than those in the standard whole body calorimeter protocol. The exercise component of the standard whole body calorimeter protocol is modest and unlikely to physically or physiologically challenge very fit individuals.

Whole body calorimetry studies and exercise studies to volitional exhaustion were undertaken on Inspire-22 participants pre- and post-expedition. The integration of whole body calorimetry with exercise testing offers a rigorous framework for quantifying physiological adaptations to polar expeditions. Together these methodologies provide mechanistic insights into the translation of metabolic perturbations into decrements in aerobic power potentially informing evidence-based strategies (e.g., nutritional requirements and recovery strategies) to preserve physiological performance and operational readiness in extreme environments. This work is the first to assess both metabolic and physiological adaptations before and after a real-world, high-altitude Antarctic expedition in a mixed-sex group. By directly comparing men and women exposed to identical extreme environmental conditions, this study provides unique insights into sex-specific responses in a genuine expedition context, surpassing the limitations of earlier single-sex or laboratory-based research.

## Materials and methods

### Inspire-22 expedition

Selection for the expedition preceded recruitment of expedition participants to the scientific study. Potential participants in the expedition were UK military tri-service volunteers and civilians with experience of extreme environments and/or expedition medicine. To be considered for the expedition, all candidates had participated in multiple rigorous outdoor training exercises either as part of the armed forces or as civilians. A medical screening was undertaken by the expedition medical officer (who was one of the expedition participants) prior to the training and evaluation session in Norway. The medical officer for the logistics company for the expedition, Antarctic Logistics & Expeditions LLC (ALE, https://antarctic-logistics.com), also undertook an independent medical screening. It is worth noting that 7 of the 9 participants were medically qualified.

The original group selected to go forward for evaluation at UK based training sessions and an extended training and evaluation session in Norway consisted of five women and six men. During these sessions, 2 women withdrew from the expedition. A replacement female volunteer was identified and accepted to be part of expedition but then withdrew from the expedition prior to the start of recruitment to the scientific study. The final list of participants for the expedition was agreed 13^th^ November 2021.

In the austral summer of 2022/2023 the nine participants (6 men and 3 women) skied 950 km from the Ronne Ice Shelf to the South pole hauling sledges weighing up to 85 kg. The expedition lasted 47 days, including 2 rest days, reaching a maximum altitude of 2850m. Details of the expedition progress and the self-selected nutrition participants consumed have been reported previously [18]

### Study participants

The nine participants in the expedition, aged 28–63 years, were invited to participate in the scientific study of the impact of the expedition on their body composition, endocrinology, metabolism, and the functionality of wearable technologies. Participation in the scientific study was voluntary and independent of participation in the expedition. All 9 participants volunteered to be participants in the scientific study and gave written informed consent prior to any data collection. Ethics approval for the study was obtained from the UK Ministry of Defence Research Ethics Committee (2125/MODREC/22) and the study was conducted in accordance with the Declaration of Helsinki. Recruitment to the study started 1st June 2022 and ended 24th October 2022 when the final participant signed the consent form prior to the pre-expedition measurements at Coventry University.

The coding for ten participants (IS22001.. IS22010) was agreed across research groups during project planning. Participant numbers were allocated sequentially at the time of the first measurement, but the last participant was allocated the last participant number identified. So participant number IS22009 was never used following the late withdrawal.

The studies reported in this paper were carried out in the UK. The period from the pre-expedition measurements to the start of the expedition was between 18 and 43 days (9–24 days before departure from the UK); and the period between the end of the study and post-expedition measurements was between 9 and 19 days (0–8 days after return to the UK). The measurement facilities had 2 whole-body calorimeters so subjects were studied in pairs whenever possible. The same protocol was used for pre- and post-expedition studies and all studies were conducted starting at the same time-of-day. The aim was to study subjects as close as possible to departure from and return to the UK with the minimum spread of times between studies. However, the timing of the studies was limited by the 2 calorimeters and determined by the availability of the participants.

### Body composition

On arrival for the whole body calorimeter studies, participants had their height and weight measured (Seca 799, Seca, UK) and then their body composition determined by Air Displacement Plethysmography (ADP) [19] using a BodPod™ 2000A (Cosmed Inc., USA). Following manufacturer’s instructions, the scales linked to the BodPod were calibrated weekly using 20 kg standards and the BodPod itself was calibrated daily using a 50L test volume. Similarly, subjects were asked to wear minimal tight-fitting garments (e.g., swimming costume and cap) and to void their bladder before measurement to minimise isothermic and water retention errors.

### Whole-body calorimeter studies

During their time in the whole body calorimeter food intake of the participants was isocaloric with a composition typical of a western diet (50% carbohydrate, 35% fat and 15% protein). The diet contained no tea, coffee, caffeinated beverages or alcohol but water and non-caffeinated herbal teas were available *ad libitum*. The calorific value of dinner on the first night was determined from the fat-free tissue weight obtained from the BodPod measurement and the calorific value of meals thereafter from the metabolic rate measured during the first night.

The participants underwent metabolic studies twice: the first in the 24 days prior to departure from the UK and the second within 8 days following return to the UK. The 36-hour measurement in the whole-body calorimeter started between 7:30 and 9:30 pm in the evening, once the body composition measurements were complete. The first evening and night were regarded as the ‘acclimatisation period’ when the participants got used to the environment and the following 24 hours starting at 07:00 the ‘reference day’.

#### Whole body calorimeter – Energy expenditure calculations.

Energy expenditure was determined by indirect calorimetry where the whole body calorimeters used were based on those described by Schoffelen et. al., [16] and the specific installation described in detail previously [4,17]. The protein metabolism was determined from the nitrogen in urine samples collected as voided [20]. Participants were encouraged to void before entering the whole body calorimeter room. Collected urine volume was measured using digital weighing scales (Salter 323, Salter, UK) and analysed enzymatically for urea and creatinine (UREA and CREAT kits, respectively, Roche, DE) using an automated clinical chemistry analyser (Cobas c702, Roche, DE). From the determined protein metabolism and the difference between the concentration of O_2_ and CO_2_ levels entering and leaving the calorimeter determined on a minute-by-minute basis, the metabolic rate of the person was calculated using standard formulae [21,22]. To maintain thermal neutrality, the environment within the chamber was controlled at a relative humidity of 57 ± 5% at a temperature of 24 ± 0.1°C during the day and 22 ± 0.1°C during the night.

**Fig 1:**
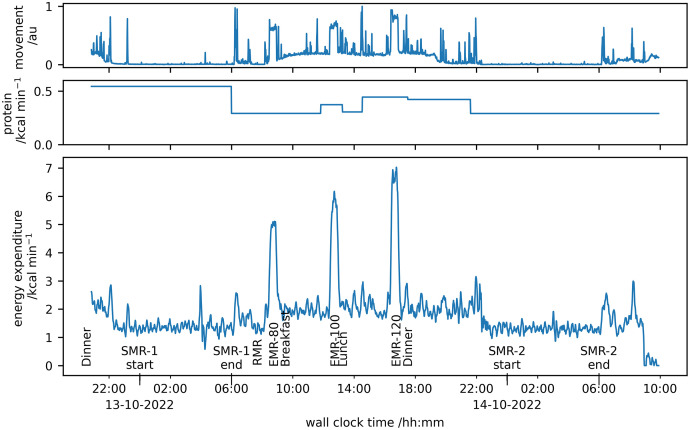
The raw data obtained from the whole-body calorimeter studies annotated with the events analysed in this paper and the times when participants were fed. The (non-protein) energy expenditure calculated from the difference in the concentrations of O2 & CO2 entering and leaving the room is shown in the lower pane, the protein energy expenditure from the urine sample analysis in the middle pane and the subject activity from the ultrasound movement sensors, scaled 0-1, is shown in the upper pane.

The study protocol is shown as part of annotated example data in Fig 1 that also shows the energy due to protein metabolism estimated from analysis of the urine samples and the participant’s movement detected by an ultrasound detector within the room (bespoke product, Maastricht Instruments, Nl).

#### Whole body calorimeter – Daily living activity measures.

The daily energy expenditure was determined as the sum of energy expenditure for the period between 07:00 after the end of the first sleep period and 07:00 after the end of the second sleep period, termed the ‘reference day’. In addition, energy expenditure and substrate utilisation data were analysed for specific events within a participants stay within the whole body calorimeter (Fig 1). Measurement of the metabolic rates for these events has been described in detail previously [3,4] but the methods are summarised here. The sleeping metabolic rate (SMR-1, SMR-2) was the average metabolic rate between 00:00 (midnight) and 06:00. The resting metabolic rate (RMR) was measured between 07:00 and 08:00 after the first sleep period. During this time, the participants were asked to lie on the bed, undertaking no activities but not going to sleep. The RMR was taken as the average of the metabolic rate values for the period 07:20–07:50. The protocol included three non-consecutive 30-minute periods of stepping exercise using a standard height exercise step (Reebok Aerobic Step, height 160 mm, Reebok, UK) at step rates of 80, 100 and 120 steps min^-1^ (denoted EMR-80. EMR-100 and EMR-120), with the step rate controlled by a simple metronome (Tempo Perfect, https://www.nch.com.au/metronome). The metabolic rate for the stepping exercises was the average for the duration of the exercise where the timing was taken from the contemporaneous laboratory records confirmed by inspection of the energy expenditure data. For each measurement analysed, the metabolic rate was normalised to remove the effect of body size.

On completion of the whole body calorimeter study, participants were fed a light breakfast and then travelled by car to Coventry University (approximately 20 minutes) to undertake the exercise studies.

### Exercise studies

Exercise studies to volitional exhaustion were carried out in an environmental chamber (4m long x 3m wide x 3m high, Sporting Edge UK ltd, https://www.sportingedgeuk.com) under normobaric normoxia (atmospheric pressure; FiO2 = 0.21, equivalent to sea level) and normobaric hypoxia (atmospheric pressure; FiO2 = 0.14, equivalent to an altitude of 3000m). It took about an hour to change the chamber from normoxic to hypoxic conditions or vice-versa. To ensure an adequate throughput, participants could either start on normoxia or hypoxia but starting condition on the pre- and post-expedition measurements was the same. The time to change the chamber conditions formed the rest-period for participants between the two conditions and during this period participants were outside the chamber under normoxic conditions.

The exercise was an incremental stepping exercise using a stepping box (step height 250 mm, Reebok UK) with each step rate duration lasting 3 minutes starting at 60 step min^-1^ and increasing by 20 step min^-1^ until volitational exhaustion was reached [23]. The step rate was timed from a software metronome (Smart Metronome and Tuner, https://ihara-product.com) running on a mobile phone with the sound amplified by a Bluetooth™ speaker. At the end of each 3 minute period a capillary blood sample was taken from the ear lobe that was subsequently analysed for glucose and lactate (Biosen C-line analyser, https://www.ekfdiagnostics.com/point-of-care/diabetes/biosen). As an alternative for those who had injuries where a stepping exercise was contra-indicated, measurements could be made on a bicycle ergometer (Lode Corival cpet, https://lode-ergometry.com/product/corival-cpet) where the crank rotations were timed at 70 min^-1^ from the metronome and resistance increased from 80W in 15W increments to produce the same pattern of intensity increase. During the exercise both an ear-lobe (Nonin 2500 palmsat, https://www.nonin.com/products/palmsat-2500) and finger pulse oximeter (Nonin WristOx2 3150, https://www.nonin.com/products/3150-usb) were used by the participant to give heart rate and oxygen saturation (SpO2). To look at the effects of sub-maximal exercise, values at 100step min^-1^ (110W from bicycle ergometry) were also analysed.

Before the start of the measurements, participants were fitted with an appropriately sized face mask (Hans Rudolph Inc. 7450 series, USA) with the straps adjusted so there were no leaks. During the exercise the mask was connected to a metabolic analyzer (Metalyzer 3B, Cortex GmbH) which recorded respiratory flows and respired O2 and CO2 on a breath-by-breath basis on a USB connected PC. Prior to measurement the Metalyzer was calibrated in accordance with the manufacturer’s instructions using a 3l flow syringe (Hans Rudolf model no. 5570) and custom proprietary gases.

At the end of the experiment the data from the Metalyzer were exported to Excel spreadsheets for analysis. Both absolute and normalized $\dot{\text{V}}\text{O2}$ values were analysed where normalization to whole body weight was used to be consistent with the exercise activities in the whole body calorimeter.

### Statistical analysis

The number of participants is small (6M, 3F) and therefore summarised data is presented as median (interquartile range). Using these limited data we want to determine: (a) whether there are differences pre- and post-expedition and (b): whether there are differences between the male and female participants. There are too few subjects for a 2-way parametric repeated measures ANOVA analysis. Therefore, the aligned rank transform (ART) technique was used to perform an ANOVA. [24]. For diurnal energetics calculations dependent variables were pre-, post-expedition and sex resulting in a 2-way ANOVA. For maximal exertion tests hypoxia/normoxia, was incorporated, resulting in 3-way ANOVA. The statistical analyses were performed in R [25]. Whilst the ART technique was designed to support analysis of a small number of subjects [24], there is a need for caution in interpreting results, particularly sex differences as the as the number of female subjects is small.

We have previously presented a detailed analysis of body composition for the INSPIRE-22 expedition [18]. The body composition data presented in this paper is that used in calculating nutrition during the whole body calorimeter experiments and for normalizing energetics and $\dot{\text{V}}\text{O2}$ measures. Therefore, no statistical analysis was performed on these data

## Results

### Body composition

**Fig 2:**
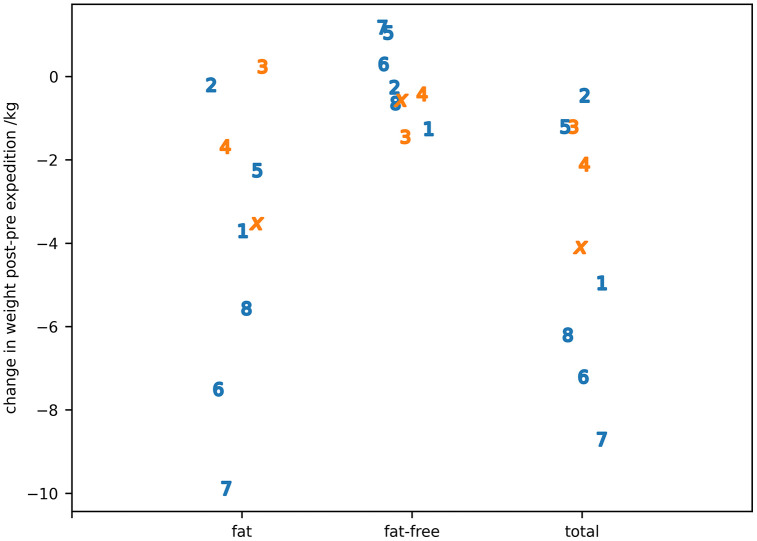
The body composition changes for individual participants post- to post-expedition. Individual participants are identified by number or letter where IS22001...IS22008 are shown as the numbers 1...8 and IS22010 as the letter ‘x’. Male participants are shown in blue, female participants in orange.

The median (IQR) values for body composition measured using the BodPod pre- and post-expedition are shown in Table 1 and the change in values for individual participants in Fig 2 which shows most of the total body weight change came from the amount of body fat present. The change in median total body weight was greater in male participants (−5.1 kg) than female participants (−2.1 kg). In terms of body composition, median fat weight loss was smaller in female participants (−2.9 kg) than male participants (−5.3 kg), but the loss of fat-free weight was greater in female participants (−1.4 kg) than male participants (−0.5 kg).

**Table 1:** Median (IQR) of the body composition of INSPIRE-22 participants.

Sex	Fat weight [kg]		Fat-free weight [kg]		Body weight [kg]	
	Pre	Post	Pre	Post	Pre	Post
Male	16.8 (1.72)	11.5 (3.39)	67.8 (0.95)	67.3 (0.71)	84.0 (3.96)	78.9 (2.83)
Female	17.0 (2.16)	14.1 (0.88)	49.9 (3.43)	48.5 (3.35)	65.2 (4.75)	63.1 (3.30)

### Daily living activities

The 24-hour ‘reference’ period contains a period of sleep (nominally 8 hours) and 3 half-hour periods of modest exercise. The remaining time is spent in sedentary activities: working, reading, watching videos and/or TV. The median (IQR) total energy expenditure during this 24 hour period for male participants is 2906 (95) kcal pre-expedition and 2840 (59) post-expedition. For female participants, the average is 2563 (181) kcal pre-expedition and 2326 (35) post-expedition.

**Fig 3:**
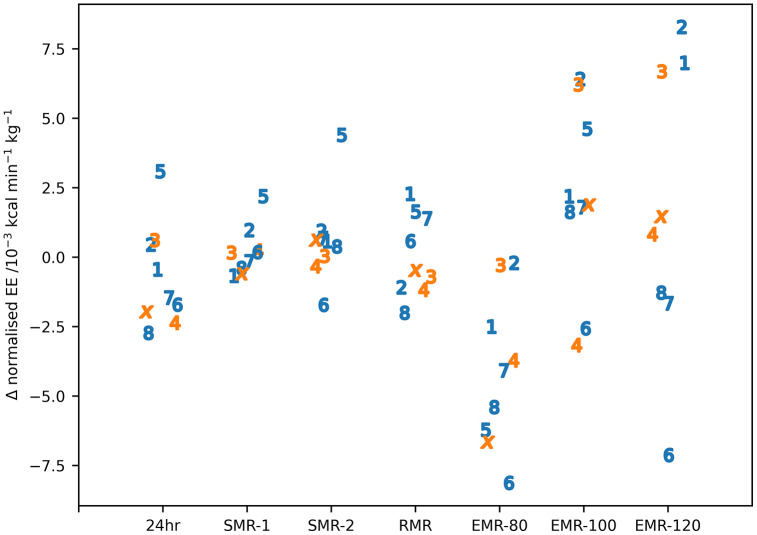
Change in energy expenditure between post- and pre-expedition measurements for individual subjects for all the activities analysed. Individual participants are identified by number or letter where IS22001...IS22008 are shown as the numbers 1...8 and IS22010 as the letter ‘x’. Male participants are shown in blue, female participants in orange. Note there is no value for EMR-120 for IS22005 as they did not perform the pre-expedition exercise.

These values of total energy consumption for the reference period are absolute values and not corrected for body size. Table 2 gives the normalized median (IQR) energy expenditure for the 24hr reference period normalized to fat free weight together with those for the DLAs during a participant’s time within the whole body calorimeter pre- and post-expedition. In addition, Fig 3 shows the change in energy expenditure for these measures for individual subjects.

**Table 2:** Median (IQR) values for the normalised energy expenditure for the activities analysed.

mnemonic	Normalisation factor	Pre-expedition /10^−3^ kcal min^-1^ kg^-1^	Post-expedition /10^−3^ kcal min^-1^ kg^-1^
24hr	Fat-free weight	29.21 (2.99)	28.86 (5.10)
Non-exercise			
SMR-1	Fat-free weight	18.53 (2.28)	19.51 (1.89)
SMR-2	Fat-free weight	25.19 (3.55)	26.14 (3.61)
RMR	Fat-free weight	27.35 (3.49)	28.72 (5.47)
Exercise			
EMR-80	Body weight	59.13 (5.91)	55.18 (6.27)
EMR-100	Body weight	66.50 (8.91)	65.97 (7.62)
EMR-120	Body weight	68.15 (10.45)	70.77 11.30)

Table 3 shows the results of the ART analysis for the daily living activity measures. The only statistically significant difference between pre- and post-expedition measurements was for the 80 step min^-1^ exercise (EMR-80) where the pre-expedition levels were higher than the post-expedition levels. It should be noted that this was the only exercise activity in the whole body calorimeter when participants were fasted. There was a consistent difference between male and female participants across all daily living activities. As noted previously, differences between male and female participants must be treated with caution because of the small number of female participants. There was no significant interaction between the dependant variables.

**Table 3:** Results of the ART analysis for energy expenditure.

mnemonic	pre|post	sex	pre|post X sex
24hr	–	p = 0.001	–
Non-exercise			
SMR-1	–	p < 0.01	–
SMR-2	–	p < 0.01	–
RMR	–	p < 0.01	–
Exercise			
EMR-80	p < 0.05	p < 0.01	–
EMR-100	–	p < 0.05	–
EMR-120	–	–	–

pre|post denotes the result for pre- and post-expedition comparison; and X denotes the interaction between dependant variables. p-values > 0.05 are shown as -.

**Fig 4:**
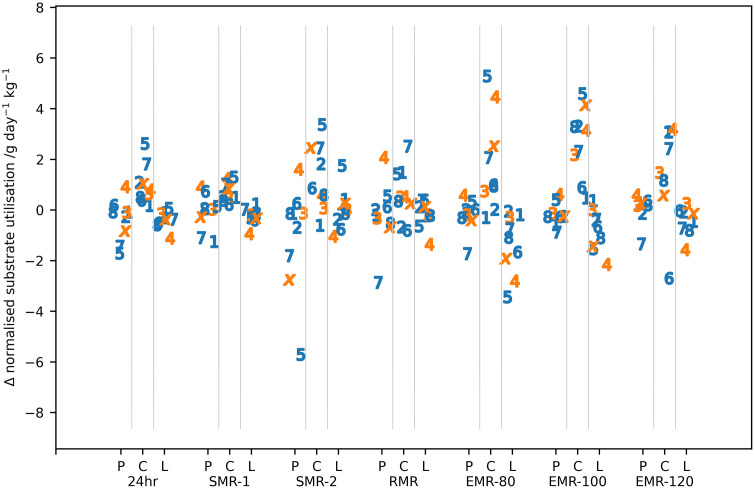
Change in normalised substrate utilisation between post- and pre-expedition measurements for individual subjects for all the activities analysed. P = protein, C = carbohydrate and L = lipid. Individual participants are identified by number or letter where IS22001…IS22008 are shown as the numbers 1...8 and IS22010 as the letter ‘x’. Male participants are shown in blue, female participants in orange.

The median (IQR) of the normalised substrate utilisation for the 24hr reference period and the events are given in Table 4 and the difference between pre- and post-expedition measurements for individual subjects in Fig 4.

**Table 4:** Median (IQR) values for the normalised substrate utilisation for the activities analysed.

	Normalization	Protein [g day^-1^ kg^-1^]		Carbohydrate [g day^-1^ kg^-1^]		Lipid [g day^-1^ kg^-1^]	
	factor	Pre	Post	Pre	Post	Pre	Post
24hr	Fat-free weight	1.61 (0.23)	1.23 (0.38)	4.67 (0.29)	5.57 (0.73)	1.47 (0.72)	0.99 (0.65)
Non-exercise							
SMR-1	Fat-free Weight	1.32 (0.52)	1.05 (0.60)	1.97 (0.87)	3.14 (1.13)	1.23 (0.29)	1.00 (0.68)
SMR-2	Fat-free Weight	2.08 (1.28)	1.79 (0.70)	3.09 (1.28)	4.34 (0.79)	1.27 (0.57)	1.23 (0.58)
RMR	Fat-free Weight	1.51 (0.71)	1.42 (0.81)	3.16 (0.79)	3.53 (0.86)	2.14 (0.80)	1.80 (0.95)
Exercise							
EMR-80	Body Weight	0.91 (0.42)	0.81 (0.38)	5.89 (0.99)	8.02 (2.09)	5.66 (0.74)	3.85 (1.45)
EMR-100	Body Weight	1.10 (0.32)	0.84 (0.17)	13.42 (4.38)	15.15 (4.96)	3.32 (1.33)	2.31 (1.55)
EMR-120	Body Weight	1.29 (0.54)	1.25 (0.39)	15.68 (4.18)	17.84 (3.15)	2.98 (1.49)	2.43 (1.16)

The results of the ART analysis applied to the substrate utilisation are shown in Table 5. There were no significant differences in normalized protein utilisation between pre- and post-expedition measurements for any of the daily living activities in the whole body calorimeter. The normalized lipid oxidation was significantly lower for the 24hr and lowest level of stepping exercise (EMR-80). Differences in carbohydrate utilisation between pre- and post-expedition measures were more complex being consistently statistically significantly higher in the post-expedition measurements with the exception of the RMR and EMR-120 measurements. A subset of participants, including both male and female participants, had much higher post-expedition carbohydrate utilisation for EMR-80 and EMR-100 and to a lesser extent for the 24hr, SMR-2 and RMR activities. Any differences between the sex of the participants must be treated with caution, but protein utilisation was consistently lower in female participants than male participants across all exercise activities and the first sleeping period, SMR-1. Normalized lipid oxidation was found to be significantly lower in female participants during the first sleeping period (SMR-1) and the highest level of stepping exercise, EMR-120. There were no significant differences with sex for normalized carbohydrate utilisation and no interaction between the dependant variables.

**Table 5:** Results of the ART analysis for substrate utilisation.

mnemonic	Protein			Carbohydrate			Lipid		
	pre|post	sex	pre|postXsex	pre|post	sex	pre|post X sex	pre|post	sex	pre|post X sex
24hr	–	–	–	p < 0.01	–	–	p < 0.05	–	–
Non-exercise									
SMR-1	–	p < 0.05	–	p < 0.001	–	–	–	p < 0.05	–
SMR-2	–	–	–	p < 0.05	–	–	–	–	–
RMR	–	–	–	–	–	–	–	–	–
Exercise									
EMR-80	–	p < 0.05	–	p < 0.05	–	–	p < 0.05	–	–
EMR-100	–	p < 0.05	–	P < 0.01	–	–	–	–	–
EMR-120	–	p < 0.05	–	–	–	–	–	p < 0.05	–

pre|post denotes the result for pre- and post-expedition comparison; and X denotes the interaction between dependant variables. p-values > 0.05 are shown as –.

### Exercise studies

**Fig 5:**
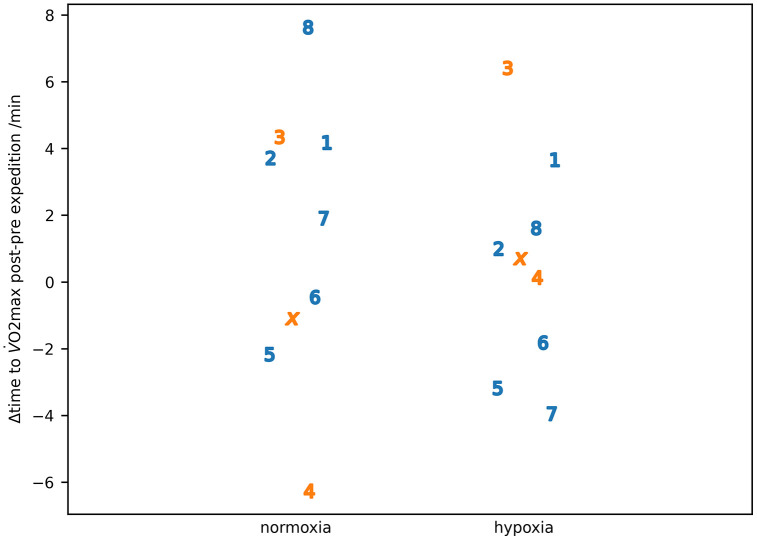
The change in time to $\dot{𝐕}𝐎2𝐦𝐚𝐱$ in normoxia and hypoxia post-pre expedition for individual participants. Participants are identified by number or letter where IS22001...IS22008 are shown as the numbers 1...8 and IS22010 as the letter ‘x’. Male participants are shown in blue, female participants in orange.

The exercise studies were designed around a stepping exercise, but the risk of exacerbating injury/weakness pre-expedition resulted in IS22006 and IS22010 undertaking the exercise on a cycle ergometer both pre- and post-expedition. Table 6 summarises values from the exercise measurements. The difference in the time to V O2max for the post-pre expedition measurements for individual subjects is shown in Fig 5.

**Table 6:** Median (IQR) of measurements at $\dot{𝐕}𝐎2𝐦𝐚𝐱$ pre- and post-expedition in normoxia and hypoxia.

	Normoxia		Hypoxia	
	pre-	post-	pre-	post-
Time to $\dot{V}O\text{2}max$ [min]	27.00 (3.75)	27.73 (7.42)	24.9 (2.78)	24.40 (6.40)
$\dot{\text{V}}\text{O2max}$ [l min^-1^]	3.85 (1.20)	3.36 (0.86)	3.20 (0.85)	2.85 (0.61.)
Normalized $\dot{\text{V}}\text{O2max}$ [ml min^-1^ kg^-1^]	46.62 (8.80)	47.54 (6.11)	38.58(5.58)	36.56 (2.70)
Blood lactate [mmol l^-1^]	8.09 (1.60)	6.05 (1.88)	7.67 (1.42)	6.20 (1.98)
Blood glucose [mmol l^-1^]	5.66 (1.10)	4.06 (1.26)	5.25 (0.51)	3.59 (0.70)
Heart rate [min^-1^]	178.62 (21.29)	169.63 (17.47)	175.17 (14.23)	169.29 (17.93)
SpO2 [%]	93.00 (3.67)	96.00 (3.17)	82.00 (4.67)	83.00 (5.33)

The normalized $\dot{\text{V}}\text{O2max}$ is normalized to whole body weight.

**Fig 6:**
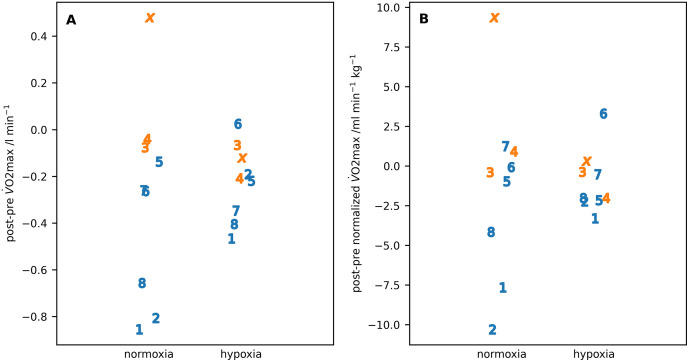
The difference in $\dot{𝐕}𝐎2$ at volitional exhaustion in normoxia and hypoxia post-pre expedition. **(A)** = absolute valuers; **(B)** = normalised to body weight. Individual participants are identified by number or letter where IS22001...IS22008 are shown as the numbers 1...8 and IS22010 as the letter ‘x’. Male participants are shown in blue, female participants in orange.

**Fig 7:**
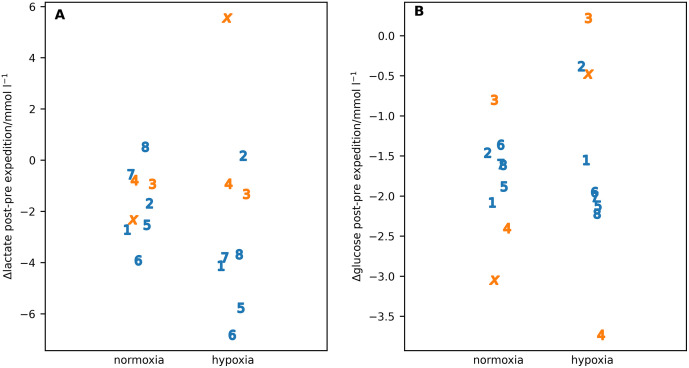
The difference in capillary (A) lactate and (B) glucose at maximum $\dot{𝐕}𝐎2$ in normoxia and hypoxia post-pre expedition. Individual participants are identified by number or letter where IS22001...IS22008 are shown as the numbers 1...8 and IS22010 as the letter ‘x’. Male participants are shown in blue, female participants in orange.

For the exercise studies, the key results were the difference post-pre expedition in oxygen utilisation at volitional exhaustion ($\dot{\text{V}}\text{O2max}$) in normoxia and hypoxia (Fig 6) and the corresponding differences in capillary glucose and lactate values (Fig 7).

The results of the ART analysis applied to the exercise data are shown in Table 7. There were significant reductions in absolute $\dot{\text{V}}\text{O2max}$ between pre- and post-expedition measurements and between normoxia and hypoxia measurements. After normalisation there was no difference between the pre- to post-expedition measurements but the difference between normoxia and hypoxia measurements remained significant. There was a significant difference with sex for the absolute measurement of $\dot{\text{V}}\text{O2max}$ and a significant interaction between the pre- and post-expedition measurements with sex for both the absolute and normalized $\dot{\text{V}}\text{O2max}$ measurements.

**Table 7:** Results of the ART analysis for exercise studies to volitional exhaustion.

	pre|post	n|h	sex	pre|post X n|h	pre|post X sex	n|h X sex
Time to $\dot{V}O\text{2}max$	–	p < 0.001	–	–	–	–
$\dot{\text{V}}\text{O2max}$	p < 0.001	p < 0.001	p < 0.05	–	p < 0.01	–
Normalized $\dot{\text{V}}\text{O2max}$		p < 0.001	–	–	P < 0.05	–
Blood lactate	p < 0.01	–	–	–	p < 0.05	p < 0.05
Blood glucose	p < 0.001	–	–	–	–	–
Heart rate	p < 0.05	–	–	–	–	–
SpO2	p < 0.05	p < 0.001	–	–	–	–

pre|post denotes the result for pre- and post-expedition comparison; n|h denotes the results for the normoxia and hypoxia comparison and X denotes the interaction between dependant variables. p-values > 0.05 are shown as –.

Summarised values for maximum heart rate and minimum SpO2 during the maximum $\dot{\text{V}}\text{O2}$ exercise are given in Table 6 and scatter plots for individual subjects in S1 and S2 Figs. Both the blood lactate and glucose were significantly lower for the post- compared with the pre-expedition measurements with lactate showing a significant interaction between both the pre- post-expedition measurements and sex and between the normixa hypoxia measurements and sex (Table 7).

**Fig 8:**
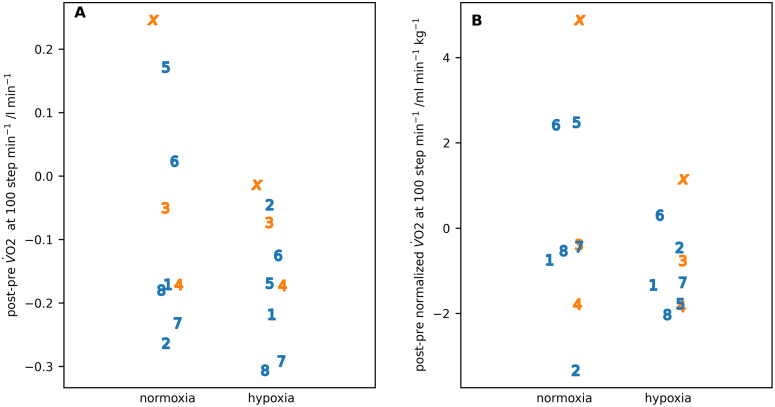
The difference in $\dot{𝐕}𝐎2$ post-pre expedition in normoxia and hypoxia at 100 step min^-1^. **(A)** = absolute values; **(B)** = normalized to whole body weight. Individual participants are identified by number or letter where IS22001...IS22008 are shown as the numbers 1...8 and IS22010 as the letter ‘x’. Male participants are shown in blue, female participants in orange.

In addition to measuring the $\dot{\text{V}}\text{O2}$ at volitional exhaustion, it was also measured at 100 steps min^-1^ (Table 8, Fig 8). There was a much larger spread of values for the difference in absolute $\dot{\text{V}}\text{O2}$ post-pre expedition for the normoxia measurements than the hypoxia measurements (Fig 8A) with female participants tending to have a smaller difference than the male participants. Table 9 gives the results for the ART analysis applied to these data

**Table 8:** Median (IQR) of measurements at 100 step min^-1^ pre- and post-expedition in normoxia and hypoxia.

	Normoxia		Hypoxia	
pre-	post-	pre-	post-
$\dot{V}O\text{2}$ [l min^-1^]	1.86 (0.27)	1.78 (0.11)	1.84 (0.20)	1.67 (0.11)
Normalized $\dot{V}O\text{2}$ [ml min^-1^ kg^-1^]	22.38 (0.46)	22.85 (3.08)	22.62 (2.33)	21.57 (2.79)
Blood lactate [mmol l^-1^]	1.08 (0.35)	1.28 (0.52)	1.45 (0.33)	1.37 (0.56)
Blood glucose [mmol l^-1^]	4.44 (0.41)	4.08 (0.19)	4.32 (0.70)	3.78 (0.37)

The normalized $\dot{\text{V}}\text{O2}$ is normalized to whole body weight.

**Table 9:** Results of the ART analysis for exercise studies at 100 step min^-1^.

	pre|post	n|h	sex	pre|post X n|h	pre|post X sex	n|h X sex
$\dot{\text{V}}\text{O2}$	p < 0.01	–	P < 0.01	–	–	–
Normalized $\dot{\text{V}}\text{O2}$	–	–	–	–	–	–
Blood lactate	–	p < 0.01	–	–	–	–
Blood glucose	–	–	–	–	–	–

pre|post denotes the result for pre- and post-expedition comparison; n|h denotes the results for the normoxia and hypoxia comparison and X denotes the interaction between dependant variables. p-values > 0.05 are shown as –.

The only significant difference between pre- and post-expedition measurements at 100 step min^-1^ was for the lower value of absolute $\dot{\text{V}}\text{O2}$ pre-expedition, a significance that disappeared after normalization. The only significant difference at 100 step min^-1^ between normoxia and hypoxia measurements was for a lower blood lactate during hypoxia. Female participants had a significantly higher $\dot{\text{V}}\text{O2}$ post-expedition when compared to male participants. As with all findings for differences between the sex of the participants, finding must be treated with caution because of the small number of female participants

**Fig 9:**
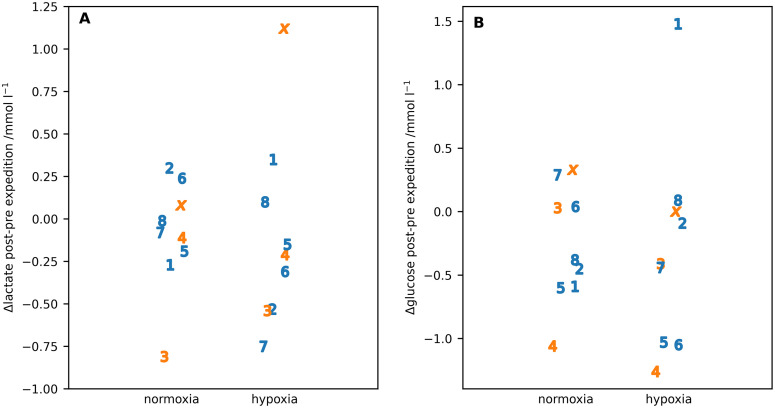
The difference in capillary (A) lactate and (B) glucose at 100 step min^-1^ in normoxia and hypoxia post-pre expedition. Individual participants are identified by number or letter where IS22001..IS22008 are shown as the numbers 1..8 and IS22010 as the letter ‘x’. Male participants are shown in blue, female participants in orange.

Figs 9A and 9B shows the post-pre expedition capillary lactate and glucose values for individual participants at 100 step min^-1^. There was a higher spread of difference in lactate values for hypoxia with IS22003 being an outlier for the difference values in normoxia and IS22010 being an outlier for the difference values in hypoxia. Neither was an outlier in the measured values. In contrast there was little difference in glucose values between normoxia and hypoxia with IS22001 being an outlier for the hypoxia measurement.

## Discussion

Values for the change in body composition reported in this paper are the result of measurements made in the UK pre- and post-expedition. A detailed study of changes in body composition together with estimates of energy expenditure during the expedition have previously been reported on INSPIRE-22 participants [18]. Body composition data included in this paper are those used to normalize the measures reported in this paper. As with participants in previous expeditions we have studied [6] the changes in energy expenditure pre- to post-expedition were small suggesting little metabolic consequence from exposure to the extreme environment of Antarctica and the high levels of physical workload. The change in median total energy expenditure during the 24hr reference period for both men and women pre- to post-expedition was consistent with the change in fat-free mass. For the majority of time during this period activities were sedentary so normalisation to fat-free body mass is appropriate. This normalisation was also used for the energy expenditure during the non-exercise activities, but normalization to total body weight was used for normalization during the exercise activities. This approach to normalization assumes body fat is inactive [26–28], which is not strictly true as there are metabolic processes which are dependent on the amount of fat present [26,27]. To include fat metabolism in the normalization requires either correlation [26] or covariance [27] measures calculated across the study population which given its inhomogeneity would not provide robust estimates of these measures. None of the study population were obese and so we have followed the advice of Arch et. al. [26] in selecting the normalization factors used in this study.

To accurately determine the metabolic rate of the DLAs, it is essential to measure energy expenditure over intervals of just a few minutes. For lipid and carbohydrate metabolism, this is readily accomplished by analysing the differences in the concentrations of O₂ and CO₂ entering and exiting the calorimeter, using established formulas [21,22]. Protein metabolism, however, which is assessed through the nitrogen content of urine, presents greater challenges. Calculating the total protein metabolised [20], or quantifying the protein content in a diet [29], typically requires urine collection over extended periods – generally 12 or 24 hours. The resulting values can be converted to an average rate of protein energy expenditure per unit time, using protein energy density and urine collection duration. In reality, this average energy expenditure is not distributed evenly throughout the urine collection period but fluctuates according to when food is consumed. Analysing urine as it is voided is an attempt to enhance the time resolution of protein metabolism measurements. It is important to recognise, though, that there are various unknown delays between absorption, liver processing, and urea nitrogen synthesis with the latter known to increase in the presence of liver disease [30]. None of the participants in this study had liver disease. There are additional underlying assumptions in estimating protein energy expenditure from urinary nitrogen, including that no protein is being used for muscle repair [20].

As in our previous analysis of sex differences in those undertaking Antarctic journeys [6] we have excluded the analysis of diet induced thermogenesis, the energy associated with digesting food [31,32], from the results reported in this paper because the group is hetrogeneous and there is no obvious method of normalizing the values obtained.

In looking in detail at the differences within the group there is a need for caution due to the small number of participants, particularly female participants. Whilst the change in median energy expenditure for the 24hr reference period for men and women was consistent with the change in median fat-free tissue mass, female participants had a significantly lower normalised energy expenditure than male participants for all three resting activities but there was no difference between pre- and post-expedition measurements. Despite this difference in normalised energy expenditure between male and female participants, there was no systematic difference in normalised utilisation for any of the substrates. There was also a significantly lower energy expenditure in the female participants for the lower two DLA exercise intensities, but not at the highest intensity. Once again there was no systematic difference in utilisation of substrates for the two lowest exercise intensities. Perhaps surprisingly, only the lowest exercise intensity showed a difference in normalised energy expenditure between pre- and post- expedition measurements. Carbohydrate utilisation was significantly higher in the post-expedition exercise for the two lower exercise intensities, but not for the higher exercise intensity. We have previously reported an increased carbohydrate utilisation in a sex-independent subset of participants post-expedition when they are fasted, but not when they are fed, particularly during low levels of physical activity [6]. In this study we also found sex-independent subsets of participants where carbohydrate utilisation was increased, most prominently in SMR-1 and EMR-100; both activities when the participants were fed. In a study where men and women wearing light clothing were exposed to air at 5^º^C men tended to have a higher carbohydrate utilisation when compared to the women but there was a large overlap of values between sexes [33].

The intensity of exercise included in the whole body calorimeter studies should not be challenging to those of normal aerobic fitness levels and thus would not be challenging to those undertaking unassisted polar travel. It is interesting to note that only the lowest intensity exercise in the whole body calorimeter showed a very significant difference in energy expenditure between pre- and post-expedition measurements and this was the only exercise undertaken when the participants were fasted. Exercise studies to volitional exhaustion are a measure of the ability to undertake high levels of exercise. Whilst there is no suggestion that the high levels of work during the expedition took participants close to volitional exhaustion, the results of the volitional exhaustion measurements are a measure of maximum capability. There was no significant difference in the time to volitional exhaustion in normoxia or hypoxia but there was a statistically significantly lower absolute $\dot{\text{V}}\text{O2max}$ post-expedition in both normoxia and hypoxia; a difference that was no longer statistically significant when $\dot{\text{V}}\text{O2max}$ values were normalized to body weight. During the expedition, there was a close to linear increase in altitude from approximately 200m to just under 3000m near the pole [18]. Exposure to altitude would have increased haematocrit and 2,3DPG [7] but the lack of difference between the pre- and post-expedition measurement of time to volitional exhaustion and $\dot{\text{V}}\text{O2max}$ suggest that these changes were at least partially, if not fully reversed at the post-expedition measurements. Evidence that at least some adaptation was still present is supported by the significant reduction in maximum heart rate during the post-expedition measurements when compared with the pre-expedition measurements and the small, but statistically significant, increase in blood oxygen saturation (SpO2) in the post-expedition measurements. It has been suggested that de-adaptation takes place over ‘a couple of weeks’ [9,10] and the studies were carried out between 9 and 19 days post expedition so de-adaptation in different participants is likely to be different. Whilst carrying out studies in the UK utilises well controlled and dedicated measurement facilities there is clearly a need to make measurements closer to the end of the expedition before any physiological changes due to the change in the daily physical activity and access to unlimited and palatable food can occur. In making this change, there is a need to recognise that the measurements will be both less accurate and less extensive.

There is a statistically significant reduction in capillary lactate post expedition in both normoxia and hypoxia coupled with a significantly reduced capillary glucose post-expedition in both normoxia and hypoxia. The former suggests that there may be changes in muscle fibre composition as a result of the daily sledge pulling regime [34]. The latter is consistent with the increase in glucose utilisation found post-expedition in the whole body calorimeter studies reported in this paper. However, care must be taken in interpreting these values as they come from capillary not systemic samples.

The results for the 100 step min^-1^ exercise in the whole body calorimeter and during the exercise studies are not comparable because the step height was higher and the study protocol was incremental in the latter with subjects starting at 60 step min^-1^. We have previously used cycle ergometers [35] and treadmills [36] for the exercise component of the pre- and post-expedition exercise testing. Neither these, nor the incremental step exercise used in this study, are representative of the effort involved in pulling sledges in Antarctica. There is a need to develop an exercise protocol that more closely reflects this and, ideally, one that can be applied close to the departure for, and return from, the expedition before de-acclimatisation to the high physical workloads can occur and before participants have unrestricted access to palatable food.

It is interesting to note that the $\dot{\text{V}}\text{O2max}$ (both absolute and normalized) and lactate show significant interactions between the sex of the participant and whether the measurements were made pre- or post-expedition. Whilst caution is needed because of the small number of women, this finding does suggest that the changes as a result of the expedition are different in men and women. Consistent with findings from a previous study of women undertaking an Antarctic traverse we found a reduction in fat-free weight at the time of the whole body calorimeter measurements [4]. However, findings from a previous study on this cohort found that fat-free weight loss tended to be less in women than in men immediately post-expedition [18]. This latter finding is consistent with findings from studies looking at energy deficit where women have been shown to conserve fat-free tissue [2] suggesting that women maybe better adapted to endurance sports although other factors may contribute to the observed difference in performance [37]. However, the findings in the current study suggest that fat-free tissue loss in women is part of post-expedition adaptation whereas the reverse is true of men. It has been suggested that the preservation of fat-free tissue in women is the result of using more fat than carbohydrate when compared to men [1]. However, we found no systematic difference in substrate utilisation between men and women but rather a subset of participants containing both sexes that used an increased carbohydrate utilisation suggesting the difference is less well defined.

We have considered acclimatisation to altitude, acclimatisation to high levels of physical effort, the final dimension of acclimatisation is to the cold. Participants experienced temperatures down to −50ºC, but for most of the expedition daily temperatures were around −15ºC. There is no reason to assume acclimatisation to a cold environment would affect the exercise studies and we saw no increase in RMR in the post-expedition measurements although a 60% increase in basal metabolic rate has been reported in those exposed to the polar environment [38]. However, participants were well equipped with appropriate clothing for the environment so for most of the time they were warm with only part of the face exposed. Anecdotally, none of the participants complained of feeling cold during the expedition, although three participants did request a higher temperature in the whole body calorimeter during the return visit. Facial exposure to cold has been shown to have minimal effect on basal metabolic rate during exercise [39].

Overall, the difference between pre- and post-expedition measurements is small confirming our previous findings [3,4] that there is minimal metabolic consequences for participants of both sexes who are appropriately trained, equipped and who have appropriate and adequate nutrition. Importantly, unlike these previous studies, this study contained both male and female participants who undertook the same expedition, so results are directly comparable, albeit for a small number of female participants. There were statistically significant differences in normalised energy expenditure between men and women for all of the activities in the whole body calorimeter except the highest levels of exercise (p < 0.05), significantly lower protein utilisation during all levels of exercise in the whole body calorimeter (p < 0.05) and significant interactions between sex and whether the measurements were made pre- or post-expedition for the normalised $\dot{\text{V}}\text{O2max}$ (p < 0.05) and lactate (p < 0.05). These results suggest that whilst the metabolic consequences are minimal for both sexes, there are differences in adaptation between male and female participants, but these can only be properly investigated by making measurements immediately before and after expeditions on a larger number of subjects, particularly female subjects.

Studies such as this allow the impact of environmental conditions on human physiology to be studied in a way that is difficult, if not impossible, to replicate in a laboratory. The necessarily small numbers of subjects in these studies gives a high level of statistical uncertainty; for INSPIRE-22, the small number of female participants is particularly challenging. The Global Polar and Altitude Metabolic Research Registry (https://www.rgs.org/in-the-field/advice-and-training/resources-for-expeditions/global-polar-and-altitude-metabolic-research-registry) has been set up to collate the environmental, expeditionary and physiological data from expeditions and individuals undertaking polar exploratory journeys where physiological measurements on participants have been done to a standard protocol. The data from INSPIRE-22 will be added to the registry. Whilst analysing data across multiple expeditions clearly presents challenges, it does have the potential to increase the statistical confidence of findings.

The work reported in this and other studies [3,4] on those undertaking Antarctic Journeys supports the need for nutrition of an appropriate quantity and composition if post expedition metabolism is to return to normal. The need for appropriate nutrition as part of managing chronic disease and post-treatment recovery whilst reducing healthcare costs is now recognised [40]. The work reported in this paper together with previous work looking at the design of diets for expeditionary travel [41] will support nutritionists designing diets for patients. It has been previously shown that the day-to-day regime for unassisted Antarctic travel is time invariant [18] and so the results from studies such as this are more readily applicable to clinical medicine for those chronically ill, whereas results from mountaineering studies [42] are more appropriate to those with acute illness. Outside healthcare, the findings also have application in planning nutrition for disaster relief.

## Supporting information

S1 FigThe Heart rate for individual participants at $\dot{𝐕}𝐎2𝐦𝐚𝐱$ in normoxia and hypoxia pre- and post-expedition. Individual participants are identified by number or letter where IS22001.IS22008 are shown as the numbers 1.8 and IS22010 as the letter ‘x’.(TIF)

S2 FigThe SpO2 for individual participants at $\dot{𝐕}𝐎2𝐦𝐚𝐱$ in normoxia and hypoxia pre- and post-expedition. Individual participants are identified by number or letter where IS22001.IS22008 are shown as the numbers 1.8 and IS22010 as the letter ‘x’.(TIF)
