# Supplementary figures and images for: Changes in metabolic energy measures for daily living activities and exercise in men and women following arduous activity in Antarctica
Source: PLoS One. 2025 Oct 31;20(10):e0335735. doi: 10.1371/journal.pone.0335735 (PMC12578249; doi:10.1371/journal.pone.0335735)

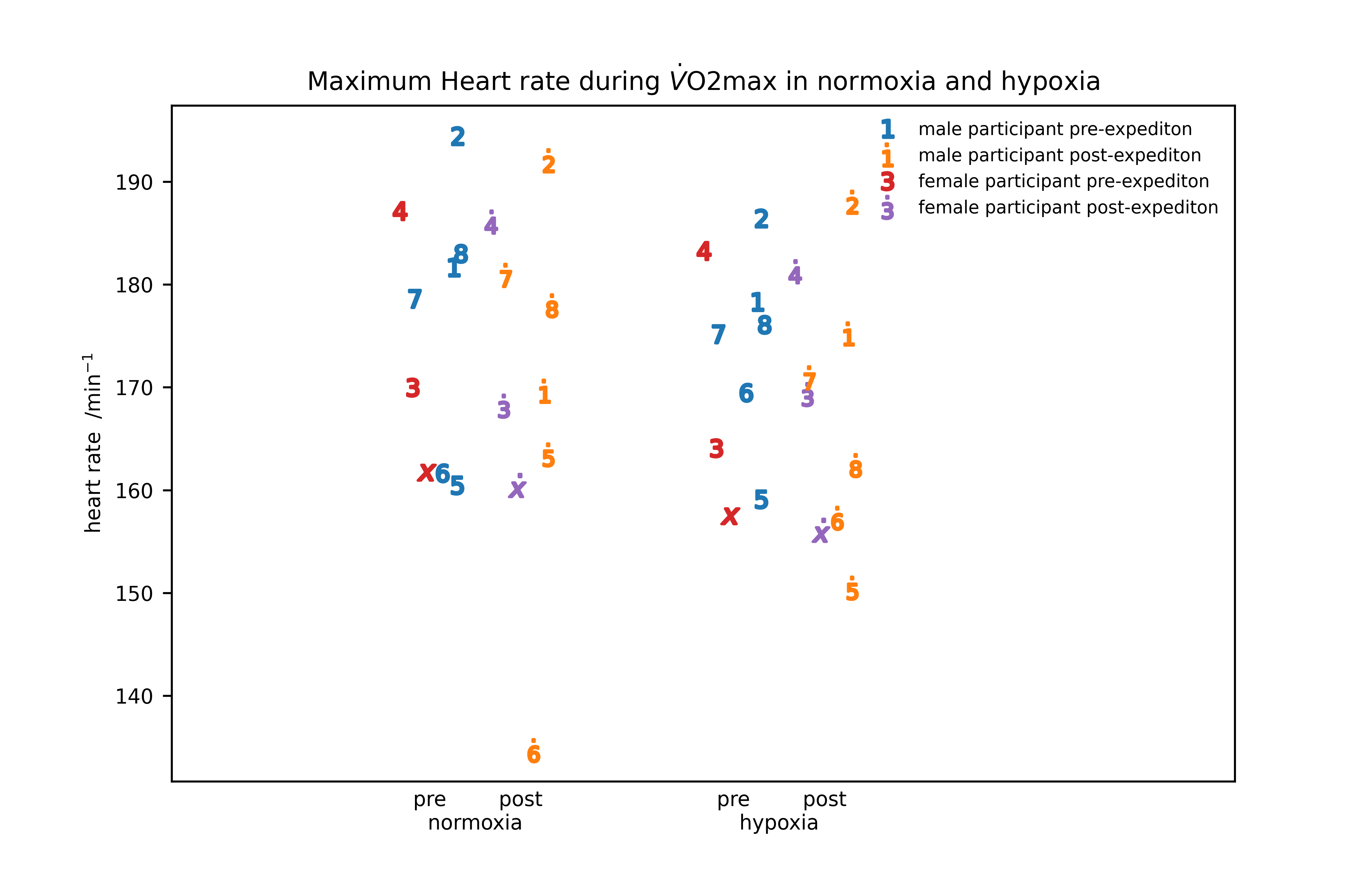

Supplement: S1 Fig — The Heart rate for individual participants at 𝐕˙𝐎2𝐦𝐚𝐱 in normoxia and hypoxia pre- and post-expedition. Individual participants are identified by number or letter where IS22001.IS22008 are shown as the numbers 1.8 and IS22010 as the letter ‘x’. (TIF) [file pone.0335735.s001.tif]

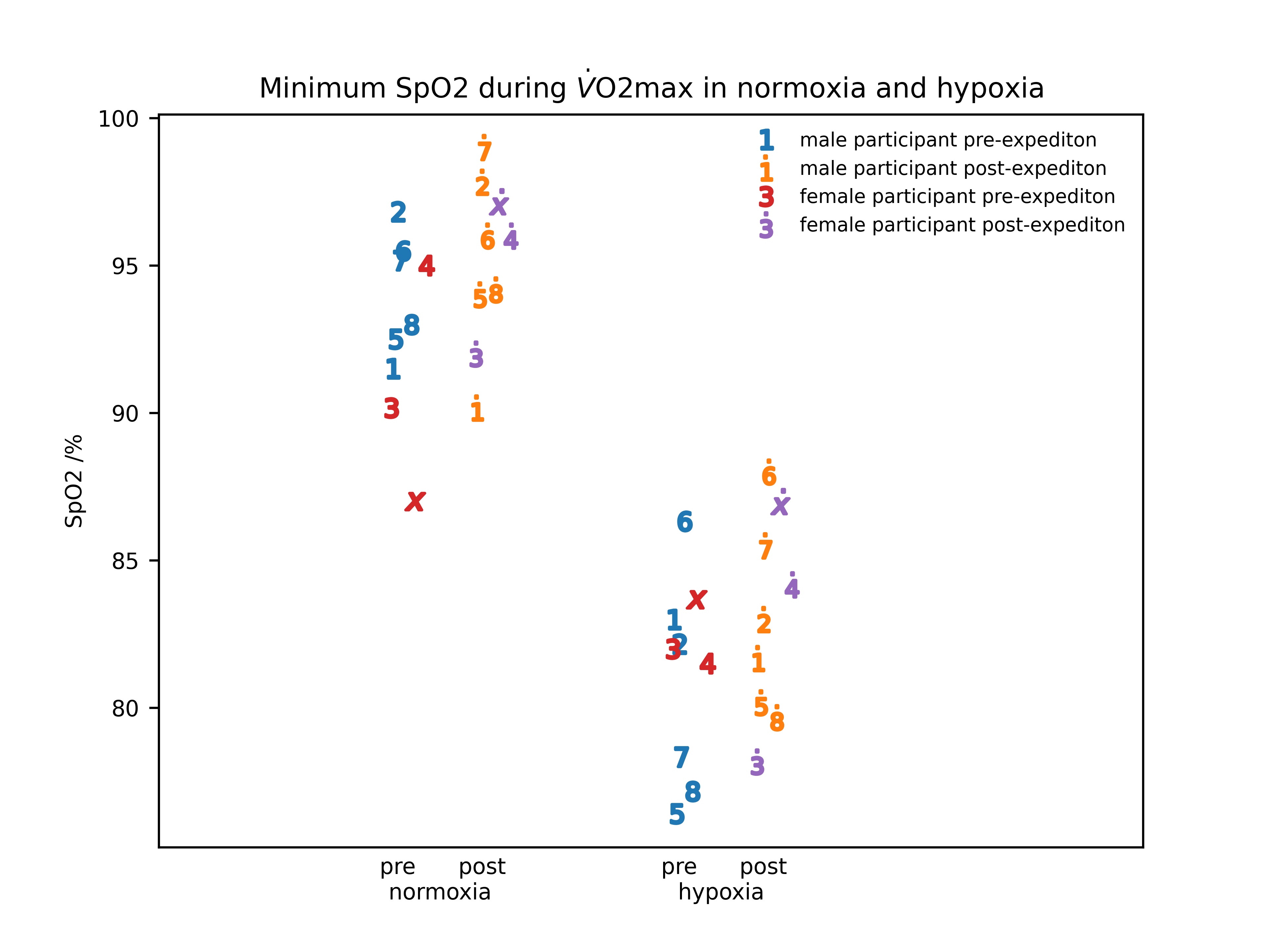

Supplement: S2 Fig — The SpO2 for individual participants at 𝐕˙𝐎2𝐦𝐚𝐱 in normoxia and hypoxia pre- and post-expedition. Individual participants are identified by number or letter where IS22001.IS22008 are shown as the numbers 1.8 and IS22010 as the letter ‘x’. (TIF) [file pone.0335735.s002.tif]
